# Toward AI Governance: Identifying Best Practices and Potential Barriers and Outcomes

**DOI:** 10.1007/s10796-022-10251-y

**Published:** 2022-04-20

**Authors:** Emmanouil Papagiannidis, Ida Merete Enholm, Chirstian Dremel, Patrick Mikalef, John Krogstie

**Affiliations:** Norwegian University of Science and Technology, Trondheim, Norway

**Keywords:** AI governance, AI data governance, AI challenges and outcomes, Performance gains, Competitive advantage

## Abstract

In recent years artificial intelligence (AI) has been seen as a technology with tremendous potential for enabling companies to gain an operational and competitive advantage. However, despite the use of AI, businesses continue to face challenges and are unable to immediately realize performance gains. Furthermore, firms need to introduce robust AI systems and mitigate AI risks, which emphasizes the importance of creating suitable AI governance practices. This study, explores how AI governance is applied to promote the development of robust AI applications that do not introduce negative effects, based on a comparative case analysis of three firms in the energy sector. The study illustrates which practices are placed to produce knowledge that assists with decision making while at the same time overcoming barriers with recommended actions leading to desired outcomes. The study contributes by exploring the main dimensions relevant to AI’s governance in organizations and by uncovering the practices that underpin them.

## Introduction

As businesses adopt Artificial Intelligence (AI), they are faced with new value propositions, but they also have to deal with new challenges, such as reducing the gap between intent and action(Amershi et al., 2019; Enholm et al., 2021; Mishra & Pani, 2020). Artificial intelligence has been perceived as a tool with which we can layer many different functions or as a solution to problems that are beyond the ability of traditional applications to solve. (Smuha, 2019). In order to gain a competitive advantage over their competitors (Raisch & Krakowski, 2021), businesses have implemented and deployed AI solutions to automate their processes, increase efficiency and reduce costs (Frank et al., 2019; Gregory et al., 2020). To achieve these goals, AI governance is essential. According to Butcher and Beridze (2019), AI governance “can be characterized as a variety of tools, solutions, and levers that influence AI development and applications”. Yet, further research is needed to better determine how AI Governance can be introduced into a company and whether AI governance can assist a company in achieving its objectives.

While AI has the potential to generate business value in terms of performance, productivity and effectiveness, it is not autonomous, as it works in concert with human capabilities (Zhang et al., 2021). Consequently, organizational capabilities are the results of combining and deploying multiple complementary resources within a firm to achieve competitive advantage (Mikalef & Gupta, 2021). When a firm optimizes its firm-level resources and adopts AI technological innovations, it can enhance its transformed projects' business value which drives business value and impacts performance (Wamba-Taguimdje et al., 2020). Simultaneously, the AI algorithms can be considered performative in the sense that they assist in decision-making, the extent to which their use can form organizational processes, or even take autonomous decisions (Faraj et al., 2018; Grønsund & Aanestad, 2020) that leads to new organization capabilities through AI. The use of AI, for instance, could create more substantial customer acquisition or higher customer lifetime value and lower operating costs or reduce credit risk.

The main goal of this work is to analyze AI governance when designing and implementing AI applications in order to achieve organizational goals. In particular, this study examines how AI Governance helps top-level managers achieve their goals by introducing robust systems that automate processes and enhancing tasks that traditionally were done by intuition or simple data analysis without negatively impacting employees. The main challenge for adopting AI in organizational operations is that AI technologies vary in scope and complexity, hindering familiarity, especially for non-technical employees (Holmstrom, 2021). Hence, it is crucial to define actions for overcoming barriers and challenges (technical and non-technical) to align AI applications to the organization’s objectives. As an example, employees might resist new technologies due to fears of being replaced by AI. Based on the results, companies will be able to gain a better understanding of how AI technologies are used, identifying focal points and mechanisms of value generation (e.g., augmentation or automation of decision-making or processes) and what challenges AI technologies present to organizations. Hence, we argue that AI value realization is not yet fully understood and called for and specific governance practices may help in doing. This study, therefore, builds on the following research questions:

- *RQ1. Which practices underpin AI Governance?*
- *RQ2. What are the antecedents and effects of AI Governance?*

To answer the research question, we collected data through a multi-case study, conducting interviews with multiple respondents within three companies in the energy sector. The interview questions focused on methodologies companies currently use, mechanisms and processes used in AI application development, the collection of data, and the consequences of AI application in decision making (AI risk). During this multi-case study, employees from various departments were interviewed, primarily the business department and the IT department since these two departments play a crucial role when developing an AI application. We also built on secondary data sources, such as reports and internal documents, which help to explore AI governance dimensions and practices as well as compare, triangulate and verify results. Among the outcomes of the study, AI was found to be most helpful for (1) reducing maintenance costs, (2) increasing flexibility and robustness of the development process, (3) improving confidence in the results and final products, and (4) gaining a competitive edge over the competition. Lastly, we proposed a model where we discussed challenges, recommended actions, and desired outcomes.

The rest of the paper is structured as follows. The subsequent section presents the background of this study and the relevant work in the domains of technology governance, and then specifically focuses on AI governance practices. Section 3 details the methodology that is applied for gathering and analyzing the data. In Sect. 4, we present each case separately followed by a cross-case analysis. The paper concludes with a discussion of the findings and limitations in Sect. 5, where we interpret and analyze the data.

## Background

### IT and Information Governance

IT governance is an area of corporate governance that falls under the responsibility of the board of executives and focuses on the implementation and transformation of IT to meet current and anticipated business and client needs and is broader than IT management, which refers to the management of existing IT services and internal supply of IT (Saunders et al., 2020; Wilkin & Chenhall, 2010). In other words, IT Governance is a formal way to align IT strategy with business strategy. Governance frameworks for IT provide a structure (who is governed, what is governed, how is governed) for ensuring that IT investments support business objectives (Tiwana et al., 2013). Through embracing IT Governance, organizations can achieve measurable results towards their strategies and goals. However, implementing a comprehensive IT governance program requires a lot of time and effort (Debreceny, 2013).

In the digital era, information governance has an even more central role, as it promotes a more purposeful path to obtaining information. (Cath, 2018). Research previously conducted in similar areas sought to answer questions like what information governance practices are firms adopting and what are the effects of information governance on performance. According to a study conducted by Intel (Tallon et al., 2013a, b), Big data governance policies achieved the main goal of maximizing business value while minimizing technical and organizational risks related to data privacy (Tallon et al., 2013a, b). Furthermore, research studies have been conducted and supported by empirical evidence on developing AI capabilities by creating a unique set of resources that can effectively leverage investments and generate business value that leads to competitive advantage (Mikalef & Gupta, 2021).

In their empirical research, Tallon and colleagues (Tallon et al., 2013a, b) discovered that Information governance is associated with a range of intermediate or process-level benefits and many of these intermediate effects could possibly affect firm-level performance. The authors suggest a need for extending structures and practices employed in IT governance and to decompose information governance into a range of structural, procedural, and relational practices. In this paper, the structural, procedural, and relational practices are used as the main dimensions to explain how to govern information and boost firm performance (Appendix).

### Governance of AI Projects

While IT governance intends to manage IT assets, hardware and software components, that assist in establishing the automation of well-defined tasks, data governance aims to manage data assets as facts having value or potential utility that are documented (Fadler & Legner, 2021). Furthermore, sophisticated forms of analytics involve artificial intelligence and automated decision-making, requiring new roles and responsibilities, but also leading to new risks. Governance should therefore not be limited to the content, but should also include its analysis, as AI should be considering a dynamic frontier of computing (Berente et al., 2021). In addition to IT and data governance, analytics governance mechanisms are needed to overcome challenges, such as the alignment among business users and analytics practitioners (Fadler & Legner, 2021). AI increasingly influences many aspects of society, from healthcare and marketing to human rights. Allowing the development of AI applications that are not under any supervision could be harmful (Chatterjee et al., 2020; Mishra & Pani, 2020); thus, it is important to promote a trustworthy AI that is lawful (complying with laws and regulations), ethical (ensuring ethical principles and values) and robust (from a technical and social perspective). For example, the use of AI in healthcare poses various issues, including a loss of privacy in health information, diminished human oversight in decision-making, and increasing prejudice across the board (Johnson et al., 2021; Trocin et al., 2021b). Governing AI projects can be interpreted differently depending on the perspective of different individuals and algorithmic management should be a concern. Because of the extent to which algorithms and the institutional frameworks allow them to get acquire management jobs to define AI's impact on key organizational processes such as delegation, coordination, and decision-making (Holmström & Hällgren, 2021).

In contrast, researchers from Microsoft (Amershi et al., 2019) approach AI governance from a technical perspective, while European Commission (EC) (Smuha, 2019) and Singapore principles approach AI governance from a human-and ethics-centric perspective. To extend this point, researchers at Microsoft (Amershi et al., 2019) have a deep focus on the technical aspects of AI. They emphasized the best practices that Microsoft teams have implemented over the years to create a united workflow that has software engineering processes and offers insights about several essential engineering challenges that an organization may face in creating large-scale AI solutions for the marketplace. According to their findings, AI governance consists of three main aspects: (1) discovering, managing, and versioning the data required for machine learning applications is more complex than a typical software application, (2) the required skills for building models and customizing them can vary based on the project, and (3) AI components might be difficult to manage if distinct modules, as well as models, exhibit non-monotonic error behavior. The European Commission’s and Singapore governments’ principles see AI governance as a way to promote Trustworthy AI through guidelines. Based on these guidelines, a framework has been created that offers guidance on fostering and securing ethical and robust AI. Further, the guidelines aim to go beyond the ethical principles by guiding how such principles can be operationalized in sociotechnical systems (Smuha, 2019). Fairness and explicability are key principles that an AI application must have, which can be achieved by governing data, reducing bias and collecting diverse data. Hence, AI can be trusted when making suggestions or taking decisions. Meanwhile, AI should be human-centric by safeguarding the well-being and safety of individuals. This calls for human oversight over AI with human agents making decisions and holding themselves accountable. As a result, it is argued that in the existing literature researchers investigate IT governance and data governance and they suggested frameworks or procedures for improving performance or minimizing risks caused by AI. There is, however, a gap in AI governance, which deals with both IT governance and data governance, and has a direct relationship with AI (Mikalef et al., 2020). Therefore, the literature would benefit from an investigation into how AI governance can increase organizational performance, while at the same time neglecting negative consequences of AI use.

### Typologies of AI Organizational Value

The value of AI in organizations varies based on the sector and the organization's activity(Collins et al., 2021). Machine learning (ML) technologies reduce the cost of repetitive, time-consuming tasks while it enhances automation and assists with predicting events or trends. But these technologies also have the ability to bring societal inequalities into organizational processes (Teodorescu et al., 2021). Lebovitz et al. (2021) discovered a knowledge gap between AI and specialists in their research, allowing managers to better understand the risks and benefits of each technology. When the underlying information is unknown, their research demonstrates the dangers of using ground truth labels objectively in ML models; thus, the organization value that AI offers has some constrains. Furthermore, in a multi-method study that comprised an analytical model, experimental testing, and a simulation study, Fügener et al. (2021) investigated how AI counsel effects complementarities between people and AI, concentrating on what humans know that an AI does not (unique human knowledge). They observed that human judgments converge on similar responses, which enhances individual accuracy. Individual unique human knowledge decreases when the group's overall individual accuracy improves (Fügener et al., 2021). Nonetheless, as revealed in a two-year ethnographic study (Van den Broek et al., 2021) when AI economic value could not be easily realized, human engagement in the development phases remained crucial. Despite the researchers' objective to keep domain experts "out of the loop," they observed that developers and experts collaborated to create a new hybrid practices that merged ML with domain experience (Van den Broek et al., 2021). Finally, when it comes to the introduction and deployment of AI, senior executives with a comprehensive understanding of the technologies have a direct positive effect on their organizations’ overall strategic direction and goals resulting in long-term economic benefits (Li et al., 2021).

## Methodology

AI Governance in both the public sector and private sector is a set of practices that still have not been consolidated. The inadequate empirical data on mechanisms and procedures that firms deploy led us to engage this research using an exploratory, comparative case study approach that boosts generalizability while at the same time giving room for extending theory via cross-case analyses (Ramesh et al., 2017). As AI will receive more attention in the following years because of the numerous challenges it poses, we sought revelatory cases that throw light on the phenomenon for the purpose of gaining a better understanding of it (Lewis et al., 2011). In addition, there is no established framework or theoretical model that is commonly accepted by the industry and describes in detail the overall governance firms should adopt. For carrying out our multiple case studies, we followed established guidelines for case study research as illustrated by Baskarada (2014), Stewart (2012) and Eisenhardt (1989). Also, we make use of the Information value chain schema to facilitate the interplay between people, processes and technologies over the information value chain, as proposed by Abbasi et al. (2016).

Trustworthiness in the evaluation process and the findings themselves were of the utmost importance; thus, we enhanced the research methodology by strengthening credibility, dependability, transferability and confirmability (Korstjens & Moser, 2018; Sikolia et al., 2013). To ensure validity in our findings, we used triangulation across multiple sources and methods through the convergence of information. In terms of transferability, the firms have common traits and operations, but they have some key differences in their business strategy. Dependability was achieved by being consistent in the analysis process and being in line with the accepted standards. Finally, confirmability was achieved by conducting interviews with different employees in the same firm who have key positions and belong to the same or different departments. What is more, data were analyzed and coded independently by three authors bringing various insights and points of view so that the authors could identify similarities and differences in their results, creating a comprehensible and coherent framework. Hence, in order to develop a theory based on empirical data, it was necessary to establish three iterations of data analysis.

### Case Selection

The selection process of the cases was conducted based on the common characteristics in respect to industry, use of AI systems, size of development teams and cultural environment. All firms operate in the same industry and have similar capabilities in terms of collecting, analyzing and interpreting data for making business decisions. The most common perspective among the selected firms is that AI must be developed, expanded and adopted in the following years as it will be crucial for gaining or maintaining their competitive advantage over rivals or new companies entering the frame and seeking a piece of the pie. Also, the nature of AI projects undertaken by firms indicates that they face similar challenges, so they require similar solutions. Comparing the selected companies is fair because (1) they are all allocated in Norway, (2) they have similar AI teams in terms of size and experience, although the size of the companies ranges, and (3) their cultural differences are limited. Therefore, choosing these three firms from the industry allows us to compare the cases for commonalities and key differences and spot how AI Governance has been implemented. Also, a generalized and standardized framework would assist companies and the state in adopting AI and planning ahead for the resources, infrastructure and necessary processes that are required. In Table 1, the cases are presented with an overview of their size, revenue and AI strategy that they follow or plan to follow in the upcoming years.

**Table 1:** Overview of companies

	Company A	Company B	Company C
Country	Norway	Norway	Norway
Sector	Energy	Energy	Energy
Employees	200	530	100
Turnover 2020	180 million dollars	260 million dollars	23 million dollars
AI Vision	Use AI to become one of the top players in the market	Use AI to increase flexibility and business capabilities	Create AI products that are customer oriented and boosts customer value
AI Technologies	Both cloud and local ML pipelines combined with intelligence dashboards – Python, Grafana	ML pipelines combined with intelligence dashboards – Python, Grafana, Power BI	ML pipelines combined with intelligence dashboards – Python, Grafana, Tableau

### Data Collection

Conducting interviews is an excellent mechanism for gathering information, especially when the researcher does not have a priori guiding theory or assumptions (Qu & Dumay, 2011). Also, interviews can be used to refine a theory or understand a phenomenon (Tallon et al., 2013a, b). As shown in the background section, previous researchers decompose information governance into a range of structural, procedural, and relational practices, which could be used as part of our baseline to understand how to build practices that enable AI Governance. A case study approach is chosen because it allows for in-depth analysis using interviews as generating method for collecting data. By exploring these data, new knowledge can be generated allowing for meaningful insights that explain similar situations (Oates, 2005). Also, the research is qualitative as it involves the use of qualitative data, which can be used to understand and explain the research question (Michael, 1997), as it involves the use of experiences, beliefs, and attitudes of the key respondents through the semi-structured interviews (Wynn & Williams, 2012).

Every case was initiated by contacting the human resources department or those who should have been able to handle this type of communication, for instance, managers. A brief introduction was sent via email to establish an understanding of the purpose of this research project and in some cases, quick telephone calls where necessary in order to provide some extra information. We described ideal candidates for interview as employees that (1) have a key position in the firm, for example, managers and leading developers, (2) have a good understanding of AI technologies and (3) have contributed to the overall development of AI either through their domain knowledge or their software development skills. A total of 15 individuals were interviewed, including both domain and technical experts who have worked in their current positions for at least one year, but have relative experience of at least five years. This means they are experienced, and they gained a solid understanding of AI development over time. Furthermore, participants shared how they understand specific issues, according to their own thoughts and in their own words (Pessoa et al., 2019) as members of either the business department or the IT department, as input from both departments is needed in order to understand how AI governance is designed. Table 2 shows information about the interviews, such as the firm candidates’ number and their current position.

**Table 2:** Responders’ role and length of interviews

Firm	Respondent	Role	Years in firm	Interview time
A	1	Chief AI officer	3	90 min
	2	AI Software Developer	3	55 min
	3	Machine Learning Engineer	3	45 min
	4	AI Software Developer	3	43 min
	5	Project Manager	4	49 min
	6	Machine Learning Engineer	3	35 min
	7	Machine Learning Engineer	3	45 min
B	1	Data Analyst	9	49 min
	2	Head of AI department	1	25 min
	3	Head of Data Analytics department	4.5	59 min
	4	Digitalization Engineer	10	55 min
	5	Head of Digitalization department	2	43 min
C	1	Data Scientist	2	65 min
	2	Head of Analytics department	3	60 min
	3	Operation Manager	3	60 min

The interviews formed on open-ended questions that led to interesting conversations, where the interviewees had the opportunity to adopt their questions based on the answers or even ask questions that were not part of the interview guidelines. Before each interview, we explained to each interviewer individually what we hope to accomplish through the interviews and what we expect to be the outcome of our research, while at the same time we encourage them to add anything they believe is relevant or that we missed during the interviews. The questions were split into three categories:

1. The business value and the organizational context where we try to see how AI grew over time.
2. The data management where the interviewees were explaining how their firm deals with data services and governance practices.
3. The control and technical aspects focused on control processes and mechanisms that ensured AI systems were acting upon set goals.

Each interview lasted approximately 55 min on average, with the range being between 25 and 90 min via Zoom, which was used to record each session and then the audio was transcribed using Otter AI. The audio files were transcribed in a verbatim way so that the text remains identical to the audio, meaning that all raw data are transparent, and the findings and results could be reproduced and tracked down rigorously. As part of the process, we had to go through the text and the audio to make sure everything was looking good since we wanted our text to match the audio and the only way to guarantee that was by checking all results manually.

In addition, we used related data publicly available on the company’s site (e.g., annual reports, vision and firm structure) because we consider them to have merit in our research. These documents served both as validations for our findings as well as information that we did not have prior to the interviews, assisting us to obtain a better understanding of the vision, objectives and regulations of each company.

### Data Analysis and Theory Building

A narrative analysis is followed for analyzing the content from the interviews as the stories and experiences shared by employees are used to answer the research questions.

As a first step, we went through the interview transcripts and commented on our initial thoughts by writing memos. Although memos are usually used at the beginning of a text analysis, we continued to use them for updating our thoughts and interpretations or even adding new ideas. The generated transcripts were imported into the software NVivo, where open and axial coding were applied, and categories were formed based on the notation process (coding). NVivo has an add-on module called “NVivo Collaboration Cloud” allowing teams to collaborate by storing projects securely in the cloud. Two of the writers had an “administrator” role while the rest had a “workspace owner” role, so it was convenient to store, upload and update our project files. Each writer was responsible for updating his content to the cloud and the administrators reviewed the changes, but not the content, in case something went entirely wrong; for example, unintentionally deletion of a file. If the administrators were satisfied, then a merge was performed and everybody could work on the updated version of the project. Backup files were part of the process in case we lost our work or needed to go back to a previous version, so at the end of each week, a backup process was in place and the files were stored independently of NVivo.

In the first iteration, we tried to identify all the concepts related to AI Governance and the adopted practices by the firms. Initially, there were 200 descriptive codes, such as, “working with domain experts” and “domain experts lead projects” but after an iteration the number was reduced to 95, since many codes were merged into a more appropriate coding name such as “domain experts take lead of a project to ensure quality of the final product”, where the combined codes become abstract.

The next logical step was to apply axial coding, where the main nodes that have been coded were procedural, relational, structural, AI development and AI challenges. In addition, comments and observations from different transcripts were combined to identify commonalities and patterns in the processes used when creating and deploying AI systems that assist firms minimize AI risks. Grouping the comments and observations, known as axial coding (Charmaz, 2014), allowed for better interpretations since the employees could refer to the same concept with similar terminology, depending based on their technical skills, knowledge, experience and position in the firm. In order to obtain a high level of confidence, researchers validated findings by examining reports, public information and presentations related to this research and focused on the AI aspects (Table 3).

**Table 3:** Nodes and possible items under each node

Dimension	Definition
Procedural	Practices associated with data migration, system messages, documentation and processes for expansion, dynamic model selection, pipeline evaluation, human and AI interaction, data quality sources
Relational	Practices that deal with employees and communicating goals, domain experts, AI education for employees
Structural	Practices associated with IT, optimization and automation, AI automation, ML pipelines, data access
AI culture	Understanding of AI capabilities, AI-phobia, Trust issues against AI
AI architecture Legal regulations Domain challenges Adoption problems Competitive Advantage Flexibility Cost maintenance Scaling up Superior AI results	Development best practices, cloud infrastructure, unified tools GDPR, legal constrains of AI use Data challenges, domain knowledge, external challenges Fear of losing position Developing unique AI strategy, keep AI knowledge in house Cloud services boost flexibility Minimize costs from various operations AI assists in scaling up without needing more resources Internal AI teams can give high value through solutions that are targeted in a specific problem and not generalized

Once all cases had been adequately analyzed, and the researchers had reached consensus, a cross-case analysis was performed. In the course of the discussion, we identified a number of patterns that were either similar or different and explored the reasons behind them through open discussion, trying to establish consistency and cohesion, arguing which interpretation seems most reasonable to our goal and how AI Governance is created among these cases and which practices companies should adopt or introduce.

## Case Analysis

### Within Case Analysis

All cases have some commonalities in their characteristics and practices. Firstly, all the cases operate in the same industry and have overlapping areas of operation. Secondly, development best practices were followed such as the use of Git, documentation and containerization platforms like Docker. Thirdly, data privacy (GDPR) is not a genuine concern (expect in the last case) since their data mainly consists of environmental data that anyone could access or buy, while legal regulations restrict them to using AI in specific areas, for instance they are not allowed to speculate on prices. Lastly, the set-up goals mainly concern reducing costs and forecasting energy demands.

#### Company A

Company A is a Norwegian company in the energy sector using environmentally friendly production and energy-related services. The main focus is on the areas of hydropower production and wind power production, meaning the center of attention is on developing renewable solutions that supports positive societal development. The company trades in different markets by forecasting how much energy is projected to be consumed each day known as intraday, while being actively involved in planning for hydropower plants. Hydropower plants are a controlled energy source that the owners can decide how much energy they want to produce, compared to wind energy that is affected by environmental variables. In this sense, optimization plays a key role.AI contributes to the reduction of predictive maintenance costs, which is challenging in Norway due to its harsh weather conditions, especially during winter.

As part of its strategy, the company developed an AI team internally and adopted or utilized cutting-edge AI technologies and techniques more extensively. A small group of recently hired developers forms the AI department and becomes part of the business development and innovation team of the company. Among the reasons for that decision was the belief that the company cannot maintain a competitive advantage without using AI in the upcoming years, and eventually, larger corporations will absorb them. The AI team brought value to the firm by forecasting energy consumption, assisting in decision-making for the end-users and automating repetitive tasks. As a result, performance was boosted and maintenance costs were down.

Control of key domain knowledge was one of the main concerns for firm. Company A did not want to give away domain knowledge to external partners, who offer specialized AI products, since they could build and sell similar AI products to their rivals:

“If we help them (the software company) develop their software, they will take this software where we provide the data, we provide domain knowledge and sell it to everyone, especially to our rivals”. (Respondent 1, Company A)

The development team aimed for automation and flexibility but they did not want to develop the entire software from scratch since it would be time-consuming to do everything. At the same time, they did not prefer to use software of other companies, so they decided to develop the intelligence that runs on top of cloud services (boosting flexibility at the same time) despite the fact that using cloud services was challenging in the beginning:

“The real challenge was not to deploy a single model but a whole cascade of models that were dynamically selected between each other”. (Respondent 3, Company A)

Standardization and unification of AI technologies was an issue because the team is consisted of people from different backgrounds and with different skills creating obstacles in AI development. The problem occurred because each member of the team had his preferences about which tools and style should use during development time, making it difficult to exchange or understand others’ code since the system was not unified. The team decided through internal workshops to unify the used tools (e.g., programming languages, databases) while creating a shared vocabulary through collaborative wiki pages:

“We were responsible for our own code. There was no code sharing, there were no shared tools that people can use amongst each other as a team, because everyone else was doing their own thing”. (Respondent 2, Company A)

In the beginning, data was exchanged through Excel files. These files were not secure, and at the same time they realized that they could not scale up, so APIs were used to replace Excel files. The necessary data was collected through vendors, so it was possible to compare data and ensure high quality outcomes for the trained models. To increase security, data access was only possible through intranet, but the company did not define clear data management roles, making the data request process time consuming:

“You're getting data from somewhere, and the data for some reason, you don't have access at that particular time. And that that's something that pops up multiple time. You can of course, try to get around, having some to wait a bit, and you know, retry”. (Respondent 4, Company A)

Multiple steps were taken into consideration to achieve robustness and reliability. To govern the process of data cleaning and model evaluation, ML pipelines were created in the cloud. This made it easier to oversee the overall process and apply quantifiable metrics on the ML results. Also, domain experts participated in the evaluation so they can provide their insights and feedback to make the model outputs reliable and trustworthy. Rather than increase profit margins, the model outputs emphasize reducing errors, because Company A places higher priority on prediction safety instead of profitability. In case of failure, local systems (ML pipelines) were ready to support decision-making, ensuring a reliable and robust system that could always generate output and assists employees with their everyday tasks:

“You still need to have an option to run them, not on the cloud solution itself, but on your local system. So basically, we do have these kinds of processes, in case something fails, because things fail much more often than you would think”. (Respondent 2, Company A)

Domain experts manage the projects as their knowledge and expertise are needed at each step of the development phase. For example, their insights could determine, which data should be needed for the machine learning models. In addition, domain experts help with the creation of meaningful dashboards that are responsible for alerting information to employees, explaining historical data and assisting in decision making for end users. At the same time, developers focused on alerting errors and failures, for instance, if a data stream stopped delivering data. Another way to ensure robust outcomes after deployment was to test the models against real-time datasets. Through This, they were able to make adjustments to the models, obtain a better understanding of the data, and improve the overall quality of the system:

“When an incident happens, usually the ones who have developed the system and some stakeholders from the rest of the organization, they sit down and sort of meet … and they questioned what happened, what was the consequences, and then the developers go into find out the reasons for that”. (Respondent 5, Company A)

Due to radical changes in processes and operations AI training for end-users was more than necessary. All these changes caused human agents to feel phobic when interacting with the machine, as they had the overall watch and check periodically that everything is in working order. From the employees’ point of view, these automations raised concerns as they saw themselves being automated and driven away from their posts, which could result in unemployment:

“People get scared of the fact that we will automate them away. So, we had a hard environment. We started talking about why we need the people here, their domain knowledge … so we had regular meetings explaining what AI can do and not”. (Respondent 1, Company A)

To summarize, company A built AI capabilities to automate procedures and assist with decision-making by using cloud services, ML pipelines and domain experts to understand data and the outcomes of models. Flexibility, productivity, and reduction of costs were the immediate effects that the company saw as positive results allowing them to remain a competitive player while achieving their set up goals of their overall AI strategy.

#### Company B

Company B is a Norwegian renewable company that focuses on customers' needs by producing and distributing clean and renewable energy. The company’s management believes that future energy consumption will differ from what it is today in many ways. Energy customers will produce their own energy and they will want to have the opportunity to combine this with smart energy solutions, meaning that the customer will more than ever be at the centre of attention where he will play a small but still significant role in the production of energy. The firm understood that the adoption of AI is vital for creating new products and services that will make them a leading provider of competence services.

Data analysts performed data surveys to evaluate which data they think to be the finest and most suited for their purposes. Within the last five years, the firm has hired a couple of analysts with machine learning experience and they have begun developing AI models in conjunction with domain experts. To build the AI capabilities data were gathered internally and externally from various vendors as it needed to verify and ensure the quality of the data since it is crucial for the AI models:

“We have a data survey, to make sure that we have the right data for what we think would do the job. And then we build the model”. (Respondent 1, Company B)

Reducing maintenance costs and errors, as well as creating flexible systems that can scale, were all top priorities. Initially, the team used cloud services, but they were not flexible enough, or at least to their liking, so they moved to influx databases that allow storing and retrieving time series data. By contrast, a containerization platform like Docker was adopted from the start to let developers to package applications into containers. Thus, these standardized executable components boosted flexibility and the cloud services were put aside. With the help of the IT department help new tools and processes were introduced to detect early problems and warnings by using different types of sensors. Based on these inputs, autoregressive (AR) models were developed to detect anomalies in the system, saving time and effort, which means fewer maintenance costs in the long run:

“We have audio surveillance, to monitor and detect early problems with just sound and then we have the AI model. It is listening to the sound and try to detect early warnings”. (Respondent 4, Company B)

“We had a cost of around two million a year and it has been reduced to around ten thousand a year, almost nothing”. (Respondent 3, Company B)

Nevertheless, it is expensive to add many features and takes a lot of time to develop. Despite the use of ML applications with neural networks, all the applications are considered to be weak AI (AI that is limited to a narrow task). Because of that the company still uses conventional and traditional ways in parallel with AI, while they plan to replace them over time in the future:

“We have used this technology started with basic AI … using more machine learning and neural networks and so on and that has only been around for two years, but it was a strategic decision”. (Respondent 5, Company B)

“It's always a question of cost them money… so that's, maybe that's why we use Excel for many processes, because it's, it's very easy to set up and when you have set up something that works, and you have to pay in order to replace it”. (Respondent 4, Company B)

Another challenge that the developers faced came from employees who refused to use the new technologies as they did not trust the results or even oppose the change. Although the AI works as assistance in most cases and helps with decision making, the employees could not accept that a new member of the firm that has no experience in their field could improve their work significantly:

“I've got some feedback from people that “you can't come here and tell me what to, how to do it. I worked here for 20 years with the same thing”. So, they are there are scared of me doing their job better, I think”. (Respondent 3, Company B)

Nevertheless, when people start using the applications, they misunderstand the AI capabilities. End-users had unrealistic expectations of what the model could or should predict, and the developers spent many hours explaining what a statistical output is and how the model actually make predictions. Furthermore, they elaborated on what is possible and what is not doable, which took a long time for the end-users to digest all these new information and the training process lasted for months.

Last but not least, the data administrator is a straightforward process because there are only two roles primarily, one administrator who can perform all actions (e.g., write and read), and one reader who can only read specific data as part of their work. This simplicity in roles and the fact that they do not deal with private data in their applications led to the decision to not have a dedicated employee responsible for data management.

To sum up, company B uses AI as a tool for prediction for identifying market opportunities and reducing maintenance costs. To accomplish this, a small team of AI developers was formed, who introduced new technologies and processes with data from various vendors. The complexity of the system was kept low to prevent high development costs while the end-users were introduced to AI capabilities to enable them to trust and adopt AI in their daily work.

#### Company C

Company C is a firm that identifies itself as climate-conscious, where they assist their customers through digital technologies to reduce energy consumption. Their services cover many aspects such as charging devices and heating in the home, which is appealing for many people as their services assist in saving a considerable amount of money every month. Company C realized that there was a big gap in the market since energy-producing companies did not offer any customized services. Hence, they decided to adopt AI practices to build the necessary capabilities to create customized applications for each client. A direct effect was that customers came with constructive feedback driving the firm to become even more efficient and building new services that were highly demanded:

“Every time a customer approaches with a question, we take those questions. And let's say a customer just comes and says like, I would like to control my water boiler at home, and I can't, and I am spending a lot of money on this. So, I would like you to improve that.” (Respondent 1, Company C)

Building these AI capabilities though would be impossible if the company did not follow best development practices. In addition, cloud services are used to cover areas that the members of the development team have no expertise or the time to develop:

“We would need to build our own data centers, which is completely out of our expertise, we would need to hire people and know how to distribute the load, then you need to secure your data etc.” (Respondent 1, Company C)

To ensure robustness, the development team has created procedures that covers extensively any AI behavior change and when the timeline that these changes are allowed to be published, for example, not before a big event, in production to avoid AI failures. AI unit tests are also in place to ensure the system's outputs are reliable. To gather the data for their AI models, Company C uses APIs from different vendors. As previously mentioned, the firm uses private data, so a dedicated team was formed to deal with privacy issues by introducing procedures during the data transformation and data storage phases:

“We have a team in the company that it's exclusively focused on privacy, and how to comply with the regulations.” (Respondent 1, Company C)

Nevertheless, data roles and data management were not always in the spotlight as almost all employees could access data since the company’s size was small. The growth in numbers led to the decision of introducing data management roles and restrictions on the data types and situations under which employees can access data. This was accomplished through data-gates where employees had to ask for permission from the supervisor of the system:

“If they need to access that data, they will need to request it from their supervisor for example. And then it depends on the type of data that you use, what data you get access to, but I would say like data scientists and developers usually we get access to basically everything because we work on everything.” (Respondent 1, Company C)

The AI applications focus on specific needs, which usually involve forecasting ancillary services, customer needs (AI assistants) and reducing maintenance costs by minimizing business risks at the same time. To ensure trustworthiness and confidence in their provided services, the team has implemented ways for explaining AI decisions (XAI) which allow customer service employees to communicate efficiently with customer requests that involved AI decisions or AI suggestions:

“The machine taking decisions and that the customer wondering why the machine took the decision and asking support for this. And then we need to tell them why the machine took this decision.” (Respondent 1, Company C)

It is worth mentioning that Company C never experienced any problems related to AI fear since all employees have a good understanding of what AI can offer and how it helps them in their everyday lives. Two could be the main reasons for that. Firstly, employees have an extensive onboarding training process and secondly, people who applied to the company are aware that the firm uses extensive AI products; thus, work candidates have prior knowledge of AI technology and AI products or are willing to embrace AI.

### Between Case Analysis

The interviewees talked about how their company transformed over the years and the necessary steps that were taken in order to expand and maintain a competitive advantage, while minimizing AI risks. In Table 4 there is a sample of the grouped observations that are generated based on the interviews.

**Table 4:** Nodes and grouped observations (sample) based on the interviews

Node	Observations	Code
Procedural	Having a backup [offline] AI model is recommended	Backup offline ML pipelines
	Use AI platforms mostly for deploying models	Build intelligence on top of external AI services
	Correct the source data not the cleaning process	Data quality sources
	understand concepts not just data	Data quality sources
	Create dashboards for monitoring actions and results	Enable human—AI interaction
	Create AI products that do one task	Create weak AI applications
	Ensemble models to maximize the output	Dynamic model selection
Relational	Onboard training processes	AI education for employees
	Operators should understand what the model is (and not) capable of predicting	AI education for employees
	Read data from different vendors to increase quality of model	Data vendors
	Domain experts take lead of a project to ensure quality of the final product	Domain experts lead projects
	Hire external consultants to predict the value of the project or help with specific cloud technologies	AI consultants
	Explain to customers AI decisions	Explainable AI
Structural	Automate operations that take place 24–7	AI Automation
	Repetitive and boring tasks should be automated	AI Automation
	AI solutions that focus on a very specific problem perform much better than generalized AI solutions	Locus of AI strategy
	Allocate required resources and create plan for AI development	Locus of AI strategy
	Access data through intranet for security reasons	Intranet data access
	No clear roles who is responsible for data management	Data ownership responsibilities
	Data transformation process has been standardized	ML pipelines

#### Procedural

As far as the procedural practices are concerned, all firms aimed to build new capabilities using external software. Algorithms, trading strategies and machine learning pipelines are developed by the internal AI teams, using platforms from third partners, keeping domain knowledge in house.

“We try to build all by ourselves. We do not want third parties to build what we can because they can use the same software for different purposes”. (Respondent 2, Company C)

For all projects, data governance, data quality and data security are common elements to ensure quality and security. All firms attempted to fix potential issues in the data sources, through data collection corrections and the use of APIs, instead of extending their cleaning process:

“We do not do much cleaning of the data; we are focusing on getting it right.” (Respondent 4, Company B)

The evaluation of ML pipelines was a continuous process that took place at different points of the pipeline for ensuring robustness and quality. The outcomes of the pipelines were AI products that are considered to be weak AI, executing singular tasks or providing with suggestions for decision making (AI assistants). Nevertheless, the end-users had to follow the AI suggestions intuitively and use their domain knowledge to fill gaps that AI was not capable of. In addition, intelligence systems should include notification systems, error detection and decision-making tools. By doing so, firms measure the credibility of their systems and evaluate the performance gained through KPIs:

“We need to always monitor the quality measures and always be on our toes and improve that.” (Respondent 4, Company B)

#### Structural

As for the structural practices, AI strategy for current or feature development projects seems to be the centre for top managers as they need to design products that focus on specific needs, while adding business value. Also, managers need to allocate the right resources and plan precisely as the costs and timelines for AI projects do not follow the usual software projects:

“We need to plan and decide how long it takes, these are the resources that we need to do it, and this is the plan, and then we will go through a decision.” (Respondent 1, Company A)

Managers had to separate the nice to have features that were often requested by either clients or employees. Otherwise, these requests could delay considerably the project and skyrocket the cost of development leading the project’s failure. A note of caution is that AI development is usually more expensive than traditional software development:

“It depends on the available resources and time; it is really costly to add a lot of AI functionality. We would definitely like to have them, but it is not feasible”. (Respondent 3, Company C)

Managers could estimate the for building a pipeline based on the project specifications. It is common in AI projects to reuse parts of one pipeline for another, which reduces the overall development time considerably. At the same time, pipelines provide confidence in the quality of the end result as the final product is robust, easily maintainable and extendable for new features.

“We have all kinds of pipeline, for example, usually, we have basic, like getting the data as a first step and we do some preprocessing. Then we do feature selection, building different models, compare the performance etc.”. (Respondent 6, Company A)

Data management practices consider mostly securing data, using secure databases and intranet access, and creating a few roles for data access, where usually there are two types of roles, (1) developers with full access and (2) end-users with access to specific data:

“There is a there is a shift now. So, if you work with data, you will get access to that data ... before everything was open …, and we needed to implement these restrictions.” (Respondent 1, Company C)

#### Relational

In all cases, domain experts were involved in all development phases for two reasons. Firstly, their domain knowledge was crucial to the success of the project, and secondly, they led the projects as project managers. Also, with the help of AI developers they built notifications systems, by declaring which notifications should be sent via email and which should be displayed in dashboards:

“If something (bad) happens, we get a warning to our email. Then we can find the bugs or look more on tools and see what happened in there and fix it.” (Respondent 6, Company A)

External AI consultants assisted only at the beginning, and they were only called on in rare cases when the development team was unsure how to proceed with a particular project:

“We had consultants for cloud services that we weren’t familiar with and for some ML optimizations”. (Respondent 3, Company B)

Lastly, establishing an AI culture inside the firm through extensive training was not an easy process, especially true for the two first cases. Employees did not trust the outcomes, sometimes they described the recommendations as naive, and most importantly, employees saw AI and automation as a way of losing their status and position. This direct threat, as they experienced it, was handled by many workshops and internal meetings.

“We explain to them that we are going to help them; we're not going to automate them away, and I talked quite a lot about this, when I explain sort of what we were doing and how it was going to work. So, taking away this fear that we were coming from the outside as aliens and our work is to identify patterns (basically) it helped a lot”. (Respondent 1, Company A)

What is more, AI teams explained what AI is all about and how it works because most employees who started using AI as assistant in their decision-making processes misunderstood very often AI’s ability to predict certain patterns (especially true when AI models were updated):

“You need to ensure that model operates in a way that the operators understand and they agree with how it was developed … allow an operator to make changes to the decision, what you often see is that the performance gets much worse.” (Respondent 6, Company A)

#### Enablers and inhibitors

Firms encounter various enablers and inhibitors when they innovate their business model. One of the main enablers for AI governance is unification in the choice of technologies and infrastructure because there are different tools for developing AI products. For example, Company A had legacy code written in different programming languages making compatibility among applications an issue. The need to unify and standardize the set of used tools was more than a necessity:

“Developers were programming in MATLAB or Python, and everyone was doing their own thing”. (Respondent 4, Company A)

Furthermore, it became essential to increase the speed of models and scale up because the company increased the amount of data while creating new intelligence based on the data. These changes were boosting efficiency and employees liked automation that lifted the heavy load of the work:

“One of the big changes and additions that everyone started programming, and automating stuff is that we went fully on cloud in all our systems, and it enabled us really be very flexible with our resources”. (Respondent 2, Company A)

AI culture promotes the acceptance of AI, meaning that employees use and trust AI. The lack of AI understanding could lead to AI phobia, which is a huge inhibitor when digital transformation process is in place. Another inhibitor could be lack of domain knowledge or lack of data for creating business intelligence. On top of that legal regulations forbid certain uses of AI, for example, the prediction of energy prices.

#### Outcomes

The outcomes were similar in all cases. That could be because their desired goals were similar. The need for reducing maintenance costs and forecasting energy consumption were the top priorities since most of the business value come from these two outcomes:

“It is similar in other industries. It is said that they are estimating, based on big data, that reduction in maintenance costs is about 20%—30%”. (Respondent 4, Company B)

Flexibility and robustness were products of the development process as their AI systems have to be able to adapt and estimate market trends. As an example is Company C, which strives to understand its customers’ habits so it can adapt to each one, while at the same time, AI decisions should be robust and non-costly for the customer to use:

“If there is a break, and someone wants to charge his car, and then start heating up… in an hour that price is high, then the cost would be pretty high… the customers is going to be angry.” (Respondent 2, Company C)

This superiority in results, boosts confidence in decisions and the potential customer value is high, especially for firms that have a more direct relationship with their clients. As a result, companies gain a significant competitive advantage over the competition as they can reduce the overall product cost and provide clients with exceptional services that adopt in their specific needs.

Table 5 shows challenges and recommended actions that firms faced and followed collectively in order to achieve desired outcomes.

**Table 5:** Challenges, recommended actions and desired outcomes

	Challenges	Recommended actions	Outcomes
Development	AI cloud is challenging to build	Offline recommendation system Develop intelligence on top of external platforms	Boost flexibility
	AI development does not follow necessarily traditional software development	Standardized executable components Unify technological tools Create shared libraries	Robustness Reduce amount of workload
	Prediction techniques vary based on sector	Allow human interaction in high uncertainty to prevent high AI bias	Robustness
	Lack of data	Choose AI algorithms based on data volume and data types Generate data from existing data Read data from different sources Buy data from vendors using APIs	Boost flexibility Robustness
	Lack of domain knowledge by AI developers	Allow domain experts to lead	Save money and time Robustness
Employees	Misunderstand of AI capabilities	AI training to understand what the models can do and what cannot do	Better communication between departments Easier adoption of AI
	Employees do not adopt AI	AI training to understand how to use the new technologies	Better communication between departments Easier adoption of AI
	Employee’s fear losing their position because of AI	AI training to explain why their expertise cannot be replaced	Better communication between departments Easier adoption of AI
	Different vocabulary for different departments	AI training to be familiar with different terms and processes Create different dashboards for different concepts	Better communication between departments Easier adoption of AI Measure performance
Value	Classical optimization tools are still better than AI models	Automate operations that 1. take place 24–7 2. there is a 1–1 correlation between workload and number of employees 3. are repetitive and boring document code and process	Save money and time Scaling up becomes easier Reduce amount of workload
	Hard to predict effort and costs	Avoid nice to have features as they will delay the whole process considerable use KPIs to quantify performance	Save money and time Scaling up becomes easier
External environment	Giving out knowledge to external partners	Develop intelligence on top of external platforms instead of using external solutions	Maintain competitive advantage
	Distance with third parties can affect development	Develop internal AI team to speed up processes considerably	AI Development is focused on your specific problem not to a generic solution maintain competitive advantage
	Legal constrains and GDPR	Create clear data management roles	Security

**Fig. 1:**
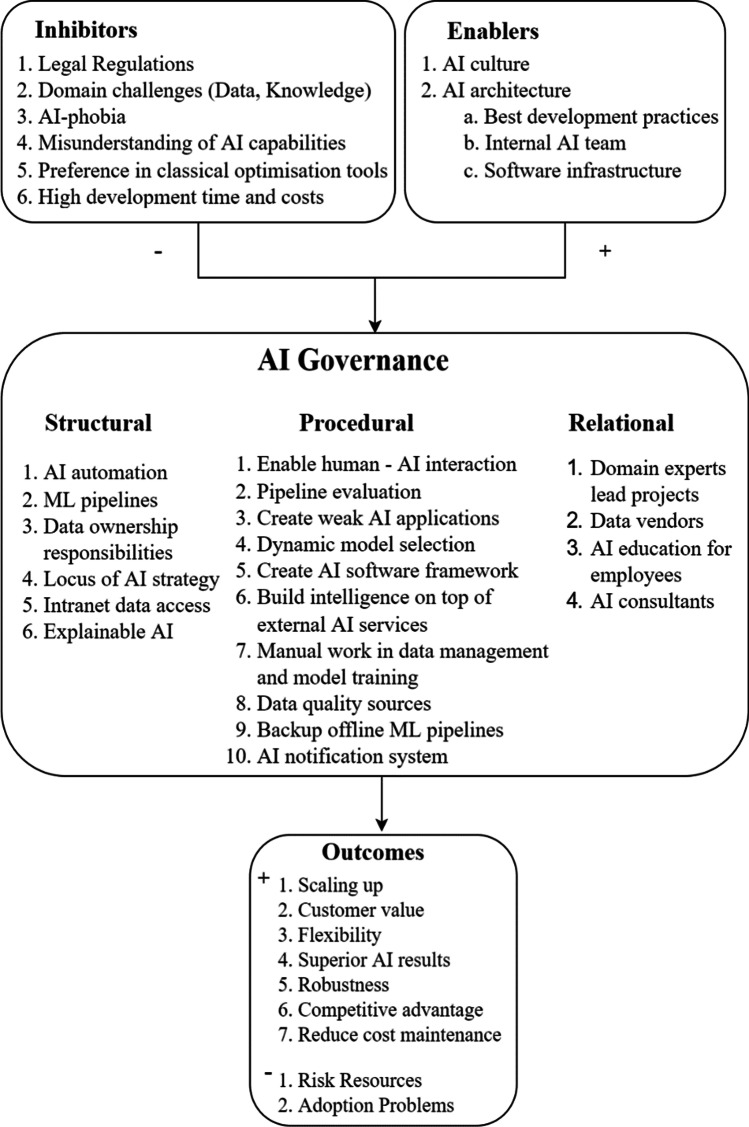
Proposed model

A proposed model is constructed based on the foregoing discussion. Our model (Fig. 1), which includes the structural, procedural, and relational components as key components, illustrates the techniques that companies have used over the last five years. Enablers include existing AI culture and architecture within a company, whereas inhibitors are mostly legal constraints, domain challenges, high development costs, and AI-phobia. Companies that seek to use AI should ensure that these problems have been examined and addressed in advance, since numerous impediments can lead to failure and waste of company resources. The model's most essential results are a competitive advantage, cost reduction, and dependable AI systems, all of which are critical to any business's success, particularly in competitive marketplaces.

## Discussion

In this study, we set out to explore the underlying activities that comprise an organization’s AI governance. Specifically, we built on the prior distinction between structural, relational, and procedural dimensions of governance in order to understand how organizations are planning around their AI deployments. Through a multi-case study of three organizations that have been using AI for several years, we conducted a series of interviews with key respondents and identified a set of activities that were relevant under each of the three dimensions, as well as challenges they faced during deployments of AI and how they managed to overcome them. Our analysis essentially points out the various obstacles that AI governance is oriented to overcoming, and the mechanisms employed to operationalize them.

Specifically, we find that the obstacles that are identified during the process of deploying AI are observable at different phases and concern different job roles. When it comes to difficult management responsibilities that a business owner must do, AI solutions can always provide a variety of responses and probabilities for each of these alternatives. However, AI lacks the ability to make decisions in specific contexts. To make the ultimate decision, a business owner or manager must employ intuition to reconcile the choices provided by AI (Kar & Kushwaha, 2021). In addition, they span various levels of analysis, from the personal, such as fear of AI and reluctance of employees to adopt it, to organizational-level ones, such as organizational directives on how to comply with laws and regulations. What is more, the study reveals not only that AI governance is a multi-faceted issue for organizations but that it spans multiple levels, therefore requiring a structured approach when it is deployed. In addition, different concerns emerge at different phases of AI projects, so AI governance also encapsulates a temporal angle in its formation and deployment.

The significance of governing AI can be critical in attaining digital innovation. The firms we looked at were leveraging AI to help them reinvent their operations. Instead of having an information collection approach, these firms followed an information analysis approach. Information analysis refers to the opportunity of developing unbiased approaches for evidence-based data analysis (Trocin et al., 2021a), where AI can foster digital process and service innovation as companies did in this study. Also, AI has the potential to foster a digital innovation process by developing new and evidence-based approaches for data collection (Mariani & Nambisan, 2021). First, it enables organizations to modify particular parameters to appeal to a wider audience when content is released online, and second, it allows them to gather online behavioral data and store it for a set period of time (e.g. one year) in accordance with GDPR regulations (Trocin et al., 2021a). It is worth mention that emotional intelligence is not part of these systems although understanding how people deal with emotional challenges is crucial for AI systems to emulate human reasoning (Luong et al., 2021). Finally, the COVID-19 pandemic has introduced new challenges and opportunities for digital transformation and innovation. For example, the United Kingdom intends to employ health information technology and execute proposals for a national learning health and care system as a result of a serious public health shock. Hence, each UK country's digital health and care strategy should be re-evaluated in light of the pandemic's lessons (Sheikh et al., 2021).

### Research Implications

This study contributes to IS literature. Despite the considerable debate in the scientific community about what is considered AI and how companies should incorporate AI in their everyday operations, we tried to understand the processes firms use to govern AI. However, not all companies have managed to build AI solutions that have had significant organizational effects and resulted in added business value. In this article, it is argued that although it is important to adopt AI, it is equally vital to create the necessary processes and mechanisms for developing and aligning AI applications with the requirements of the business environment. One of the main challenges we identify is that AI governance requires continuous adaptation and modification as new data emerges or conditions change, for instance how employees perceive AI. Thus, there is a form of ephemerality which places an increased focus on establishing processes, mechanisms, and structures to ensure that it is functioning as required and that it aligns well with the goals of the organization.

Furthermore, there is a multitude of angles that a firm can approach AI governance; for instance, companies in this study tried to create ML pipelines and interactive dashboards, but not all of them had a real focus on explainability of the results since they are still in early stages and focus on parts that they believe are more urgent. In the industry there is a recent article by Microsoft, which focuses primarily on the technical aspects of workflow implementation, outlining the key phases in the lifecycle of machine learning applications (Amershi et al., 2019). Yet, this research concentrates on the development challenges and the practical solutions a firm could follow to build an AI through solid and effective organizational practices. In this sense, AI governance in this article is not seen as a process but as a set of important aspects that need to be considered when designing and deploying practices and mechanisms, in order to ensure that the main challenges are overcome successfully and that AI applications are operating as planned. Our proposed model suggests that although there are inhibitors and barriers and despite the different ways of approaching AI governance, it offers positive outcomes, if best practices are followed, and this study identified specific procedural, structural and relational components that are necessary for achieving this.

Our exploratory work opens up a discussion about what AI governance comprises of, and how it can be dimensionilized. Furthermore, it explores the link between the challenges such governance practices help overcome, and the actors and practices they involve. This stream of research is particularly important in the value-generation of AI-based applications, as it paints a more detailed about how relative resources are leveraged in the quest for business value (Mikalef et al., 2019). In addition, the work sheds some light on the process-view of AI deployments by opening up the dialogue about the different phases of AI deployments and the unique challenges faced within each of these.

### Practical Implications

Based on the findings, a firm needs to incorporate new procedures when adopting AI in order to maintain an advantage over the competition and boost efficiency. A unified system is required for building AI pipelines, which is consistent with the tools that developers use. Hence, the system will be more robust as it will be easier to maintain and improve different components of the system. In addition, managers should create procedures that employees are aware of and follow and give clear guidelines; otherwise, time and resources might be wasted, which could be invested in other projects that would add more business value.

Firms should use AI for automating tasks that are repetitive, which is appreciated by employees since they do not want to do monotonous work, but at the same time managers should have extended conversations with employees of other departments ensuring them that AI will not replace them (AI education). This could be crucial for the company’s internal stability as people might lose trust in the leadership, they might leave the company taking their expertise with them or resist using new technologies and try to undermine the value of AI.

Lastly, firms can use dashboards as an effective way to allow communication between human and machine. Dashboards are a great information management tool that is used to track KPIs, metrics, and other essential data points relevant to a business. That way the black-box nature of models and AI in general can be less problematic because the use of data visualizations simplifies complex data sets and provides end-users useful information that can affect business performance. In other words, humans will be able to evaluate results and detect any outliers or anomalies in processed data. This in turn facilitates greater transparency and a more direct way of revising the models used to analyze data.

### Limitations and Future Research

In the current work, we investigate how to govern AI, which practices should be adopted and how to minimize AI risks. However, there are certain limitations that characterize this research. First, the data are collected through interviews with companies that do not require extensive use of sensitive data; thus, there might be bias in our data or provide an incomplete picture of the entire challenges around relevant practices. Second, while we conducted several interviews with key employees within the organizations, our data collection was based on a snapshot in time and may not accurately reflect the complete breadth of practices. Lastly, all cases are from the same sector. Hence, generalizability could be an issue that should be taken into consideration.

As future research, it would be interesting to gather more empirical data through interviews, from firms that belong to different sectors, and theorize the notion of AI governance from a positivist perspective, which could be tested with empirical data on the antecedents and their effects. It would also be beneficial for the field to know which resources firms deploy most in order to achieve their organizational goals and how they govern these resources to boost their performance, and how AI governance practices impact specific types of resources.

## Appendix

### Interview Guidelines

**Introduction**

1. What is your current role and background in the company?

**Business value/organizational context**

1. Could you mention briefly the history behind AI use in your company? How long it took you to adopt AI (timeline)?
2. How did (i) the use of AI grow over time, (ii) how did the AI team grow over time (iii) how did the value/effectiveness of AI grow over time?
3. Are there any changes brought by AI that you did not anticipate?
4. Do you plan to use AI in other aspects of your company?
5. Do you prioritize reducing risks or potential margin profits and why?

**Dealing with Data**

1. Do you deal with Data Privacy?
  1. If yes, how do you do that?
  2. If not, why not?
2. How do you handle data?
3. Could you describe the cleaning process?
4. Do you use cloud services?
  1. If yes, then what type of server do you have?
  2. What about external services like Azure?
5. How are your organization’s models audited for security or privacy vulnerabilities?
6. Do you follow any best practices for Trustworthy AI?
  1. If yes, which one?
  2. If not, why not?
7. Which people have access to your AI features? Describe the main roles.
8. Have you established any governance practices? For example, have you defined roles and responsibilities?
9. Who is in charge of the data management and what were the requirements for that position?
10. Have you quantified decision bias in your company’s model predictions?
11. Could you describe the infrastructure of your system?

**Control and Technical Aspects**

1. What types of data do you collect? How do you ensure to use data and AI algorithms such that they are in line with your organizational objectives?
2. Are there any procedures or processes for managing the data you use in your organization (for AI purposes)?
3. Where is data stored? How is it shared etc.? (In what cloud service are data stored?)
4. Do you specify, monitor and evaluate the (i) behavior and (ii) outcomes of your AI systems and potentially the combination with human decision-makers? Which actions are taken upon this?
5. Which control processes and mechanisms are in place to ensure that AI systems are acting upon your set goals? Does this differ depending on the use cases?
6. What processes do you have to ensure robustness?
7. Do you develop any kind of internal AI software framework?
8. What development practices do you follow as a team?
9. Did you try to incorporate external AI software?
10. What practices have you adopted to ensure scalability?
